# Supplementary material for: The association of sun exposure, ultraviolet radiation effects and other risk factors for pterygium (the SURE RISK for pterygium study) in geographically diverse adult (≥40 years) rural populations of India -3rd report of the ICMR-EYE SEE study group
Source: PLoS One. 2022 Jul 21;17(7):e0270065. doi: 10.1371/journal.pone.0270065 (PMC9302760; doi:10.1371/journal.pone.0270065)
Supplement: Attachment — Submitted filename: Response to Reviewers.docx [file pone.0270065.s002.docx]

**Response to Reviewers**

We thank the editorial committee and the reviewers for their expert comments and suggestions. We have revised the manuscript as per their recommendations. Please find below point-by-point responses to the reviewers and editor’s suggestions.

**Editor’s Comments**

**Q1.** Please ensure that your manuscript meets PLOS ONE's style requirements, including those for file naming.

**A1.** We thank the editor for the comment. The manuscript has been updated as per the Journal’s style requirements.

**Q2.** In your Data Availability statement, you have not specified where the minimal data set underlying the results described in your manuscript can be found. PLOS defines a study's minimal data set as the underlying data used to reach the conclusions drawn in the manuscript and any additional data required to replicate the reported study findings in their entirety.

**A2.** We thank the editor for the comment. We have now uploaded the data to the Figshare repository, available with the following citation:

*Tandon, Radhika; Vashist, Praveen; Gupta, Noopur; Gupta, Vivek; Yadav, Saumya; Deka, Dipali; et al. (2022): ICMR_Pterygium_Study_Data.csv. figshare. Dataset.*  [*https://doi.org/10.6084/m9.figshare.19626003.v1*](https://doi.org/10.6084/m9.figshare.19626003.v1)

**Q3.** Thank you for stating the following in the Financial support Section of your manuscript:

“Indian Council of Medical Research (ICMR), India provided funding for this research project (Grant No 68/4/2009-NCD-1). The funding source had no role in the study design, in the collection, analysis and interpretation of data, writing of the report; and in the decision to submit the article for publication.” We note that you have provided additional information within the Financial support  Section that is not currently declared in your Funding Statement. Please note that funding information should not appear in other areas of your manuscript. We will only publish funding information present in the Funding Statement section of the online submission form. Please remove any funding-related text from the manuscript and let us know how you would like to update your Funding Statement. Currently, your Funding Statement reads as follows: “RT (Grant No 68/4/2009-NCD-1) Indian Council of Medical Research”.

Please include your amended statements within your cover letter; we will change the online submission form on your behalf.

**A3.** We thank the editor for pointing this out. The financial support section has been removed from the manuscript **(Page no: 19)** and the updated Financial statement has been included in the cover letter.

**Q4.** Thank you for stating the following financial disclosure:

“RT (Grant No 68/4/2009-NCD-1) Indian Council of Medical Research”. Please state what role the funders took in the study.  If the funders had no role, please state: "The funders had no role in study design, data collection and analysis, decision to publish, or preparation of the manuscript." If this statement is not correct you must amend it as needed. Please include this amended Role of Funder statement in your cover letter; we will change the online submission form on your behalf.

**A4.** We thank the editor for the comment. We have included an updated Financial statement along with the role of the funder statement in the cover letter.

**Q5.** We note that you have stated that you will provide repository information for your data at acceptance. Should your manuscript be accepted for publication, we will hold it until you provide the relevant accession numbers or DOIs necessary to access your data. If you wish to make changes to your Data Availability statement, please describe these changes in your cover letter and we will update your Data Availability statement to reflect the information you provide.

**A5.** We thank the editor for pointing this out. The data has now been submitted to the Figshare repository. The information is available with the following citation:

*Tandon, Radhika; Vashist, Praveen; Gupta, Noopur; Gupta, Vivek; Yadav, Saumya; Deka, Dipali; et al. (2022): ICMR_Pterygium_Study_Data.csv. figshare. Dataset.*  [*https://doi.org/10.6084/m9.figshare.19626003.v1*](https://doi.org/10.6084/m9.figshare.19626003.v1)

We have also updated our data availability statement in the cover letter.

**Q6.** Please review your reference list to ensure that it is complete and correct. If you have cited papers that have been retracted, please include the rationale for doing so in the manuscript text, or remove these references and replace them with relevant current references. Any changes to the reference list should be mentioned in the rebuttal letter that accompanies your revised manuscript. If you need to cite a retracted article, indicate the article’s retracted status in the References list and also include a citation and full reference for the retraction notice.

**A6.** We thank the editor for the comment. We have reviewed the cited references and made the required corrections. There was an error in the citation of references 12 and 13 in the manuscript which has been corrected. **Page no: 4, Line no: 76,89; Page no: 5 Line no: 123**

**Q7.**The methodology can be condensed by using appropriate references so that it is crisp and less time consuming for readers to go to the crux of the study.

**A7.** We thank the editor for the comment. The methodology has been modified accordingly and appropriate references have been added. **Page no:5, Line no: 105**

**Reviewer’s Comments**

**Q1.** The lifetime cumulative effect of sun exposure was calculated with the help of satellite-based data for environmental UV exposure. How relevant and accurate is the data? Is the data for entire lifetime was calculated based on the current location where the study took place? Also AOD values and meteorological data was used in the study from three mentioned locations. Are those accounted at the time of examination? Or any time period? How the author will justify it based on the participants occupation location? As some might have different workplace where they might be exposed more than that particular location where the measurement was done. It will be better if explained elaborately.

**A1.** We thank the reviewer for bringing up this important point. The estimation of lifetime effective sun exposure was not satellite-based but was calculated for every participant in the study using the following formula, based on the Melbourne visual impairment project model:

*Lifetime Effective Sun Exposure = Σ [Daily hours of sun exposure without headgear + (Daily hours of sun exposure using headgear x protection factor)] x 365 x Number of years.*

The study location has no role in the determination of lifetime cumulative effective sun exposure. We can assure the reviewer that this data is reliable and the Melbourne model has been successfully used by several studies for calculating lifetime ocular sun exposure.

Satellite-based data for the entire region was used for the measurements of AOD, UVA and UVB flux at the three study locations for the study period i.e. between 2010 and 2016. In addition, meteorological data for humidity, precipitation, temperature, wind speed, and air pollutants were also obtained for the three locations for the study period.

We agree with the reviewer that the topics of lifetime cumulative exposure and climatic parameters need an elaborate explanation. These topics have been discussed in detail in our previous publications (mentioned below). To keep the manuscript concise and easy to read we decided not to discuss it in depth in the current manuscript and have provided appropriate references for the same.

1. Vashist P, Tandon R, Murthy GVS, Barua CK, Deka D, Singh S, Gupta V, Gupta N, Wadhwani M, Singh R, Vishwanath K; ICMR-EYE SEE Study Group. Association of cataract and sun exposure in geographically diverse populations of India: The CASE study. First Report of the ICMR-EYE SEE Study Group. PLoS One. 2020 Jan 23;15(1):e0227868. doi: 10.1371/journal.pone.0227868.
2. Tandon R, Vashist P, Gupta N, Gupta V, Sahay P, Deka D, Singh S, Vishwanath K, Murthy GVS. Association of dry eye disease and sun exposure in geographically diverse adult (≥40 years) populations of India: The SEED (sun exposure, environment and dry eye disease) study - Second report of the ICMR-EYE SEE study group. Ocul Surf. 2020 Oct;18(4):718-730. doi: 10.1016/j.jtos.2020.07.016.

**Q2**. Is there any association between pterygium and the type of work the participants does?

**A2.** We thank the reviewer for this comment. In this study, the occupation of the participants was classified primarily as indoors and outdoors. This is pertinent that there might be some variation in the specific job that an individual does but our predominant focus was to gather information on whether the individual had sunlight exposure during his working hours. What mattered was overall time spent outdoors and not only related to occupation. In the overall population, we did not find any association between pterygium and whether the occupation of participants involved indoor or outdoor activity. **Page no: 9, Table 2.**

**Q3.** Why indoor smoke exposure was considered? Does it include passive smokers as well?

**A3.** We thank the reviewer for highlighting this important point. The indoor smoke exposure due to the use of biomass fuel is a major contributor to indoor (household) pollution and is associated with a number of acquired systemic diseases. For this study, indoor smoke exposure was defined as a lifetime history of use of biomass fuels (coal, dung-cakes, wood) in the kitchen. A positive association of indoor smoke exposure with cataract and dry eye disease has been found in our previous analyses. Our aim was to find any such possible association between pterygium and indoor smoke exposure.

In order to account for the similar nature of exposure and mechanism of action on the eyes, passive smokers were included in the group “smokers” along with active smokers and not with the participants exposed to indoor smoke.
